# Supplementary material for: Two-photon calcium imaging of the medial prefrontal cortex and hippocampus without cortical invasion
Source: eLife. 2017 Sep 25;6:e26839. doi: 10.7554/eLife.26839 (PMC5643091; doi:10.7554/eLife.26839)
Supplement: Transparent reporting form [file elife-26839-transrepform.docx]

***eLife’s* transparent reporting form**

We encourage authors to provide detailed information *within their submission* to facilitate the interpretation and replication of experiments. If you have any questions, please contact us: [editorial@elifesciences.org](mailto:editorial@elifesciences.org).

**Sample-size estimation**

- You should state whether an appropriate sample size was computed when the study was being designed
- You should state the statistical method of sample size computation and any required assumptions
- If no explicit power analysis was used, you should describe how you decided what sample (replicate) size (number) to use

Please outline where this information can be found within the submission (e.g., page numbers or figure legends), or explain why this information doesn’t apply to your submission:

No statistical methods were used to pre-determine sample sizes but our sample sizes are similar to those reported in a previous publication (e.g. Huber et al., Nature 484, 2012; Harvey et al., Nature 484, 2012; Hasegawa et al., Cell Reports 18, 2017). Additionally, in several analyses we did, to avoid detecting false statistical significances by using a relatively large-sample size for statistical analysis, we randomly chose a small amount of data from the pooled dataset and conducted statistical analysis on them. The sample size for each analysis is indicated in the corresponding part in the main text, figure legends and Materials and Methods section.

**Replicates**

- You should report how often each experiment was performed
- You should include a definition of biological versus technical replication
- The data obtained should be provided and sufficient information should be provided to indicate the number of independent biological and/or technical replicates
- If you encountered any outliers, you should describe how these were handled
- Criteria for exclusion/inclusion of data should be clearly stated
- High-throughput sequence data should be uploaded before submission, with a private link for reviewers provided (these are available from both GEO and ArrayExpress)

Please outline where this information can be found within the submission (e.g., page numbers or figure legends), or explain why this information doesn’t apply to your submission:

The numbers of experimental repetitions and animals are stated in each figure legend and the Materials and Methods section. No outlier exclusion was performed.

**Statistical reporting**

- Statistical analysis methods should be described and justified
- Raw data should be presented in figures whenever informative to do so (typically when N per group is less than 10)
- For each experiment, you should identify the statistical tests used, exact values of N, definitions of center, methods of multiple test correction, and dispersion and precision measures (e.g., mean, median, SD, SEM, confidence intervals; and, for the major substantive results, a measure of effect size (e.g., Pearson's r, Cohen's d)
- Report exact p-values wherever possible alongside the summary statistics and 95% confidence intervals. These should be reported for all key questions and not only when the p-value is less than 0.05.

The statistical test for each experiment is clearly defined in the Results section or figure legends. Procedures of resampling and permutation tests are described in Data analysis subsection of the Materials and Methods section.

Please outline where this information can be found within the submission (e.g., page numbers or figure legends), or explain why this information doesn’t apply to your submission:

(For large datasets, or papers with a very large number of statistical tests, you may upload a single table file with tests, Ns, etc., with reference to page numbers in the manuscript.)

**Additional data files (“source data”)**

- We encourage you to upload relevant additional data files, such as numerical data that are represented as a graph in a figure, or as a summary table
- Where provided, these should be in the most useful format, and they can be uploaded as “Source data” files linked to a main figure or table
- Include model definition files including the full list of parameters used
- Include code used for data analysis (e.g., R, MatLab)
- Avoid stating that data files are “available upon request”

Please indicate the figures or tables for which source data files have been provided:

N/A
